# Reply to Saleh, C.; Budincevic, H. Interleukin-6, Tocilizumab and Atherosclerosis. Comment on “Gerasimova et al. Interleukin-6: Cardiovascular Aspects of Long-Term Cytokine Suppression in Patients with Rheumatoid Arthritis. *Int. J. Mol. Sci.* 2024, *25*, 12425”

**DOI:** 10.3390/ijms26072953

**Published:** 2025-03-25

**Authors:** Elena Gerasimova, Tatiana V. Popkova, Irina G. Kirillova, Daria Gerasimova, Evgenii L. Nasonov, Aleksandr M. Lila

**Affiliations:** 1 Department of Systemic Rheumatic Diseases, V.A. Nasonova Research Institute of Rheumatology, Kashirskoe Shosse, 115522 Moscow, Russia; popkovatv@mail.ru (T.V.P.); dr.i.kirillova@yandex.ru (I.G.K.); nasonov@irramn.ru (E.L.N.); amlila@mail.ru (A.M.L.); 2 Chair of Organization and Economy of Pharmacy, Institute of Pharmacy, A.P. Nelyubina, I.M. Sechenov First Moscow State Medical University (Sechenov University), 96k1 Ave. Vernadsky, 119526 Moscow, Russia; gerasimova_d_a@staff.sechenov.ru; 3 Russian Medical Academy of Continuous Professional Education of the Ministry of Healthcare of the Russian Federation, Build 1, 2/1 Barrikadnaya St., 125993 Moscow, Russia

We thank the authors for their interest in our study [1,2].

This study investigated the effect of 265 weeks of therapy with the IL-6 receptor blocker tocilizumab (TCZ) on cardiovascular risk factors and potential association with atherosclerosis in patients with RA. Because atherosclerosis in patients with RA often develops subclinically, the early identification of high-risk individuals is key to preventing its clinical consequences.

We used the detection of carotid atherosclerotic plaques (ASPs) as a surrogate marker of preclinical atherosclerosis. The use of carotid intima-media thickness (cIMT) for this purpose in patients with RA is problematic because of the inability to differentiate subclinical vasculitis and/or wall hypertrophy from subclinical atherosclerosis [3].

Our study showed that there was initially a high prevalence of carotid ASPs among patients with RA. One ASP was identified in 20 cases (54%) and two ASPs were identified in 4 (11%). New ASPs were identified in three (8%) patients during the study. During the 5-year follow-up period, there was a slight increase in bilateral maximum cIMT (0.85 mm before treatment, 1.05 mm after 265 weeks).

We consider your remarks to be fair.

Errors in the presentation of the study methods have been corrected, including a detailed protocol for cIMT measurement below.

According to established protocols, cIMT was measured in plaque-free segments of common carotid arteries (CCAs). The examination included scanning of the left and right CCAs, the carotid bifurcation area, and the external and internal carotid arteries with a focus on the far wall of the artery in three fixed projections—anterior, lateral, and posterior. Intima-media layer thickness was determined 10 mm proximal to the CCA bifurcation, according to recommended guidelines [4]. The cIMT was measured as the distance from the leading edge of the first echogenic area to the leading edge of the second echogenic area. One researcher was responsible for all cIMT measurements throughout the study. The entire procedure was recorded on digital media for subsequent analysis using the M’Ath software package for Windows 8 and 10 (Metris, SRL, Villers-Bretonneux, France). he average of three measurements (in the anterior, posterior, and lateral projections) was considered the integral value of the mean cIMT. Measurements were performed three times in one cardiac cycle, including the arithmetic mean for each side and the arithmetic mean between the cIMT of the right and left OCA (mean cIMT).

The measurement of cIMT was synchronized with the cardiac cycle. The cIMT measurements were performed at end-diastole under minimal distending pressure, so the carotid artery images were synchronized with the R wave of the ECG. This was part of our methodology, and we deemed it unnecessary to specify it separately in the article.

We used values from two time points to accumulate the averages, potentially missing relevant variations between visits.

We sought to reduce the number of issues raised by the writers of the letter by following a standard protocol used in studies, with rigorous training and quality control measures [5]. Most importantly, baseline scans were reread simultaneously with follow-up scans by a single interpreter, which was essential for ASP surveillance.

The 2007 recommendations of the European Society of Hypertension (ESH), the European Society of Cardiology (ESC) on arterial hypertension, and the Russian Society of Arterial Hypertension, when identifying damage to target organs, also define the upper limit of the normal сIMT as 0.9 mm when detecting target organ damage.

In the publication, we discussed the maximum сIMT, which is not the same as the mean сIMT. We hasten to correct an inaccuracy pointed out by the authors of the letter. Below, in Table 1, we present new data on the mean cIMT, which was calculated as the maximum value of the mean cIMT.

The bilateral mean cIMT value was not significantly higher in patients with RA by the end of the study (0.69 vs. 0.75 mm, respectively; *p* = 0.09) (Table 1). These data are not shown in the manuscript [1].

In our study, a mean cIMT ≥ 0.9 mm was initially found in two (4%) patients and was more frequent after 265 weeks (9 (20%) patients, *p* = 0.049).

Although these results were obtained in relation to cIMT, in a similar analysis, using the presence of ASPs as a reference, we found no differences before and after 265 weeks of TCZ therapy. This is because formation of ASPs represents a more advanced stage of the atherosclerosis process than an increase in cIMT.

It is well known that cIMT increases with age, even in the absence of overt or occult atherosclerosis, as a result of thickening of both the intimal and medial layers [5]. Aging of the arterial wall, body size, and muscularity may influence the cIMT score.

An increased cIMT may also be a consequence of subclinical vasculitis or wall hypertrophy, as has been shown in patients with RA [3].

In our study, the increase in TIM was 0.06 mm over 5 years (or 0.012 mm/year). Our results are in agreement with the data of В. Burggraaf et al. [6], showing an increase in IMT by 0.043 mm over three years (or 0.014 mm/year).

The development of cardiovascular events has been shown to be more predictive of ASP in several studies and cIMT in others [5,7]. According to scientific evidence, increased cIMT is associated with the development of atherosclerosis in arterial vessels [8] and with the risk of future cardiovascular events [9,10]. A meta-analysis showed that a 0.10 mm increase in cIMT was associated with an 18% increased risk of stroke and a 15% increased risk of myocardial infarction [11].

We assume that the increase in the number of patients with RA with cIMT > 0.9 mm after 265 weeks was related to the older age of the patients studied. The absence of significant increase in the mean cIMT was associated with the suppression of subclinical inflammation of the vascular wall on the background of RA activity reduction and the normalization of lipid metabolism in the patients receiving statins.

## Figures and Tables

**Table 1:** Dynamics of mean cIMT in patients with RA (*n* = 45).

Parameter	Before Treatment	After 260 Weeks
Mean cIMT left, mm	0.70 [0.68; 0.91]	0.76 [0.68; 0.92]
Mean cIMT right, mm	0.68 [0.60; 0.89]	0.74 [0.61; 0.94]

cIMT—the carotid intima-media thickness.
